# Accuracy of Digital Impressions and Fitness of Single Crowns Based on Digital Impressions

**DOI:** 10.3390/ma8073945

**Published:** 2015-06-29

**Authors:** Xin Yang, Pin Lv, Yihong Liu, Wenjie Si, Hailan Feng

**Affiliations:** 1 Department of Prosthodontics, Peking University School and Hospital of Stomatology, Beijing 100081, China; E-Mails: yangxinlv@bjmu.edu.cn (X.Y.); lvpinyang@bjmu.edu.cn (P.L.); 2 Department of General dentistry, Peking University School and Hospital of Stomatology, Beijing 100081, China; 3 School of Material Science and Engineering, Tsinghua University, Beijing 100081, China; E-Mail: wjsi@tsinghua.edu.cn

**Keywords:** digital impressions, accuracy, fitness

## Abstract

In this study, the accuracy (precision and trueness) of digital impressions and the fitness of single crowns manufactured based on digital impressions were evaluated. #14-17 epoxy resin dentitions were made, while full-crown preparations of extracted natural teeth were embedded at #16. (1) To assess precision, deviations among repeated scan models made by intraoral scanner TRIOS and MHT and model scanner D700 and inEos were calculated through best-fit algorithm and three-dimensional (3D) comparison. Root mean square (RMS) and color-coded difference images were offered. (2) To assess trueness, micro computed tomography (micro-CT) was used to get the reference model (REF). Deviations between REF and repeated scan models (from (1)) were calculated. (3) To assess fitness, single crowns were manufactured based on TRIOS, MHT, D700 and inEos scan models. The adhesive gaps were evaluated under stereomicroscope after cross-sectioned. Digital impressions showed lower precision and better trueness. Except for MHT, the means of RMS for precision were lower than 10 μm. Digital impressions showed better internal fitness. Fitness of single crowns based on digital impressions was up to clinical standard. Digital impressions could be an alternative method for single crowns manufacturing.

## 1. Introduction

In 1971, Duret first put forward the concept of applying computer-aided design and computer-aided manufacturing (CAD/CAM) in dentistry [1]. Since then, the application of CAD/CAM has increased significantly [2] and offered an opportunity to make use of industrially prefabricated materials with refined composition and microstructure. Laboratory digitizing technique has been the necessary access to the CAD/CAM workflow, including stone cast scanning and conventional impression scanning.

High precision impression materials, like polyether, vinyl polyether silicone, and vinyl polysiloxane in combination with stone casts, could offer an acceptable procedure to transfer clinical situation to the dental laboratory [3,4,5,6,7]. However, several potential errors may occur during this procedure, such as deficiencies on impressions and stone casts; volume changes of impressions and stone casts during disinfection, storage and transportation; partial or extensive separation of the impression material from the tray; the effects of surface moisture on detail reproductions of the impressions [3,4,5,6]. In addition, conventional impression-taking may cause patients’ discomfort like nausea, unpleasant taste, breathing difficulty and teeth sensitivity [8]. Digital impression technique is also an access to the CAD/CAM workflow that abutment and arch information are captured directly in patients’ mouth by intraoral scanning. Intraoral scanning has been reported to give higher efficiency [8,9], increase patient comfort [8], and facilitate the examination of preparation for clinicians and data storage. Communications between professionals as well as between dentists and patients are becoming more convenient due to intraoral scanning. So the digital impression technique is winning patients’ and doctors’ preference [8,9].

A number of intraoral scanning systems have been developed and introduced to dental markets. Currently two separate genres of digital impression systems exist. CEREC AC with Bluecam (Sirona Dental Systems, Bensheim, Germany) based on Stripe-light projection generates a virtual model instantly and allows clinicians to design their restorations chair-side at the same time [10], which makes chair-side esthetic prostheses possible when combined with a chair-side milling machine. The system iTero (Cadent, Carlstadt, NJ, USA) based on parallel confocal imaging technology, Lava C.O.S. (3M ESPE, Seefeld, Germany) based on active wavefront sampling, three-dimensional (3D) progress (MHT, Verona, Italy) based on confocal microscopy and Moiré effect, and TRIOS (3Shape, Copenhagen, Denmark) based on ultrafast optical sectioning concentrate mainly on image acquisition and virtual model formation [10]. Data are then transferred to the dental laboratories or factories, where restorations are designed, further processed, and finally manufactured.

Digital impression is proposed to be an alternative to conventional impression technique [1,11,12]. However, the scanning quality of digital impression was strongly influenced by patient-related factors, such as patient movement, limited intraoral space, intraoral humidity and saliva flow [11]. Tooth shape was also an important factor related to precision [13]. Therefore, as the first step of CAD/CAM, how accurate digital impressions are compared to conventional procedures is an essential issue that needs to be considered.

To describe accuracy, the parameters of “trueness” and “precision” are applied. Trueness refers to the closeness of agreement between the arithmetic mean of a large number of test results and the true or accepted reference value; precision refers to the closeness of agreement between test results, according to ISO 5725-1:1994 [14]. For 3D models, appropriate software is often used to analyze the agreement between two surface datasets by superimposition [10,12]. Usually, the software uses best-fit algorithms to export the root mean square (RMS) and 3D comparison to export color-coded difference image. Peters *et al.* [15] proposed that a RMS value of less than 10 μm is considered to be an excellent fit for virtual models. The accuracy of digital impressions of single crowns, bridges, full arch and implant by several scanners had been discussed, while the results were varied. For full-arch scanning, the precision of iTero, CEREC AC and Lava C.O.S was reported 50 μm [11], 30.9 μm and 60.1 μm [16]. Trueness of Lava Chairside Oral Scanner was reported to be 15–30.8 μm [12,17]. The accuracy of iTero, CEREC AC, Lave C.O.S and E4D system were also compared *in vitro* assessing the influence of different test materials and coating thicknesses in a recent study [18]. Detecting the fitness of the final respective restoration is another way to show accuracy [12]. The marginal and internal fit of a restoration is regarded relevant for the longevity of a restoration [10,19,20]. Although threshold values vary from 50 to 200 μm, Mclean [21] showed that crown marginal discrepancies ranging up to 120 μm were clinically acceptable, which was mostly adopted in studies. Although several studies had been done in the last three years, the accuracy of TRIOS and MHT intraoral scanners were not mentioned.

The objective of this *in vitro* study was to evaluate the accuracy of digital impressions acquired by TRIOS and 3D progress intraoral scanners, to detect the fitness of single crowns based on digital impressions and to compare with stone cast images from D700 (3Shape) and inEos (Sirona Dental Systems) model scanners. The tested null hypothesis was that the accuracy of digital impressions is same with stone cast images, and the fitness of final restorations based on digital impressions and stone cast images have no significant difference.

## 2. Experimental Work

### 2.1. Model Fabrication and Grouping

The study protocol was reviewed and approved by the Ethical Committee of Peking University School and Hospital of Stomatology (No: PKUSSIRB-201417113).

Twenty-one intact molars were collected from the clinic of Peking University School and Hospital of Stomatology, which were extracted because of severe chronic periodontitis. Teeth preparations of these teeth were done according to the clinical preparation principles, with 1.5 mm occlusal reduction and 1.0 mm width round-shoulder margin. Each tooth preparation was embedded in epoxy resin with silicone rubber gums to form a #14-17 artificial dentition (#16 was the preparation). The artificial dentitions were randomly divided into five groups. Group 1 contained only one artificial dentition, which was used to detect the precision and trueness. Groups 2–5 contained five artificial dentitions for each, which were used to detect the fitness of the final restorations. The artificial dentitions were preserved in distilled water during the test. Conventional impressions of vinyl polysiloxane impression material (Aquasil Ultra and Aquasil Soft Putty, Densply, York, PA, USA) were taken under the manufacturer’s recommendations using individual autopolymerizing resin trays [22], and stone casts were made with type IV gypsum (Heraeus, Hanau, Germany) 24-hours later.

A mandible and a maxilla (without #14-17) stone cast were also made with type IV gypsum using standard rubber molds (NISSIN, Yokohama, Japan).

### 2.2. Precision

The artificial dentition from Group 1 was fixed in a dental simulator (NISSIN) together with the stone cast of mandibular dentition. Digital impressions of #14-17 were obtained by TRIOS and MHT intraoral scanners 10 times each. All the data were exported in Standard Tesselation Language (STL) format for further calculation (TRIOS 1-10 and MHT 1-10).

The stone cast of Group 1 was scanned 10 times by D700 and inEos model scanners respectively, and STL format files were exported (D700 1-10, inEos 1-10).

All STL datasets were imported into the inspection software (Geomagic Qualify 12.0, Geomagic, Morrisville, NC, USA). The images outside the finishing line of preparations (#16) were deleted by software to insure precise superimposition. The 10 datasets for each scanner were aligned pairwise by best-fit algorithm (The one with smaller number was served as control surface, *n* = 45) to calculate the RMS of Euclidean distances of aligned points (*n* = 1500) from the two tested datasets for quantitative analysis. 3D comparisons were conducted to export the color-coded difference images for qualitative description of deviations distribution. Thus quantitative analysis combined with qualitative description was helped to make more comprehensive and reliable conclusions [23].

Statistic analysis was performed with SPSS 20.0 (IBM SPSS Inc., Chicago, IL, USA). The Kruskal-Wallis test was applied to analyze the differences of RMS. The level of significance was set at 0.05. Maximum, mean, median and interquartile range deviations of RMS were calculated to assess errors.

### 2.3. Trueness

The artificial dentition from Group 1 was digitized by micro computed tomography (micro-CT) system (Inveon Acquisition Workplace) (Siemens; Knoxville, TN, USA) to obtain the reference data. Parameters were as follows: 360° rotation, 2000 ms exposure time, 80 kV voltage, 500 μA current, and effective pixel size 9.21 μm. The volume data of micro-CT scanning was then post-processed by Mimics 10.0 software (Materialise, Leuven, Belgium) to generate STL file. The image outside the finishing line of preparation (#16) was deleted by Geomagic Qualify 12.0 software. Then the final STL dataset was exported and defined as the reference value (REF).

The STL files of TRIOS 1-10, MHT 1-10, D700 1-10, inEos 1-10 were imported into Geomagic Qualify 12.0 with REF served as control surface. The deviations (RMS) between REF and each dataset were calculated. Color-coded difference images were generated for quantitative analyses of deviations distribution.

SPSS 20.0 was used to calculate the means and standard deviations (SD) of each scanner and to compare the differences between scanners using a one-way analysis of variance (ANOVA) in combination with least significant difference (LSD) post hoc test (*p* = 0.05).

Geomagic Qualify 12.0 was also applied to evaluate deviations among TRIOS, MHT, D700 and inEos groups.

### 2.4. Marginal and Internal Fit

The dentitions from Groups 2 and 3 were fixed in the dental simulator, TRIOS and MHT intraoral scanners were used to get the digital impressions respectively. Otherwise, the stone casts of Groups 4 and 5 were scanned by D700 and inEos model scanners respectively. The final restorations were designed by DentalDesigner (3Shape) for Groups 2–4, while inLab software 4.0 (Sirona Dental Systems) for Group 5. The simulated cement thickness was set at 60 μm, 1 mm away from the margin. The final crowns were machined from a polymethyl methacrylate (PMMA) blank with the milling machine (ZenoTEC T1, Wieland Dental, Pforzheim, Germany).

Each crown was cemented to the original tooth preparation with resin cement (Bisco; Schaumburg, IL, USA) according to the instruction under pressure of 20 N for 3 min. The crowns and preparations were embedded in autopolymerizing resin (NISSIN) individually and sectioned longitudinally in the buccopalatal and mesiodistal directions with a linear precision saw (Isomet 1000, Buehler, Lake Bluff, IL, USA). The sectioned surfaces were polished by silicon carbide paper (NOON, Shanghai, China) of #160, #320, #600, #1200, and #2000 step by step. Then, the thickness of the cement layer on the section was measured under stereomicroscope (EC3, Leica Biosystems, Wetzlar, Germany) at × 40 magnification. Then, the marginal gap (MG) and internal gap of occlusal (IGO)/axial surfaces (IGA) (three adjacent points for IGO and IGA) were estimated according to the terminology reported by Holmes *et al.* [24]. A single investigator conducted all the measurements. The mean value of IG (IGO and IGA) was calculated.

SPSS 20.0 was used to calculate the mean and SD of MG, IGO and IGA for each group. One-way ANOVA in combined with LSD post hoc test (*p* = 0.05) was used to compare the fitness of different groups.

## 3. Results and Discussion

Patient-related factors influence the accuracy of digital impressions [11]. In this vitro study, a dental simulator was used to mimic the limited scanning space, reducing the freedom of placement of scanning head, so the difficulties in digital impression-taking procedures were increased. Although a dental simulator cannot reproduce the complexity of oral environment, such as oral humidity, sudden movement of patients’ jaw and head, blocking of tongue, and light condition, repeated scanning were required in this study. It was hard for the gingiva to stay still after gingival displacement for a long time. So a simulated oral environment was adopted instead of *in vivo*. The difference in gingival reflection might also be a limitation of *in vitro* study. Most of the studies with respect to the accuracy of scanners have been carried out on standardized abutments fabricated from resin or metal alloys [25], while in this study, natural teeth instead of the same-sized dies were adopted to relate the results to the clinical conditions.

As pointed out by Gimenez *et al.* [17], the achievable accuracy of digital impressions also depends heavily on operator skills. Therefore, only one experienced operator performed all the digital and traditional impressions.

In this study, datasets were evaluated by superimposition to assess precision and trueness. This superimposition was performed by best fit alignment, which was already used in several other studies as an approach for 3D dataset comparison, showed a reproducibility of less than 1 μm [10,12] for the procedure of cutting and alignment of two datasets.

### 3.1. Precision

Maximum, mean, median, and interquartile range deviations of RMS for each group were listed in Table 1. Kolmogorov-Smirnov test revealed a non-normal distribution of RMS from each group. Therefore, the different groups were compared using the Kruskal-Wallis test followed by all pairwise multiple comparisons (α = 0.05). Statistical significant differences were exhibited between scanners (*p* < 0.001). Model scanners (D700 and inEos) showed better precision than the intraoral scanners (TRIOS and MHT), which was consistent with conclusions drew by Tabea *et al.* [11] about iTero and D250. The data acquisition procedure of intraoral scanners is done incrementally, while it is a relatively continuously static procedure of model scanners. Patients’ movement, limited intraoral space, and vibration of the scanning head [11] might influence the precision of digital impression.

The results of this study showed that the mean value and median of MHT were a little higher than 10 μm, while mean values and medians of the other scanners were lower than 10 μm. According to Peters’ proposal [15], excellent agreement was found between repeated images of TRIOS, D700 and inEos. The precision observed in this study was much better than previous studies [11,16], probably because that quadrant digitization had lower precision compared to single-tooth digitization and deviations were accumulated during data increment.

The results of quantitative analyses showed that TRIOS had acceptable precision for single crowns, although a little worse than model scanners. However, the precision of MHT needs to be improved.

**Figure 1:**
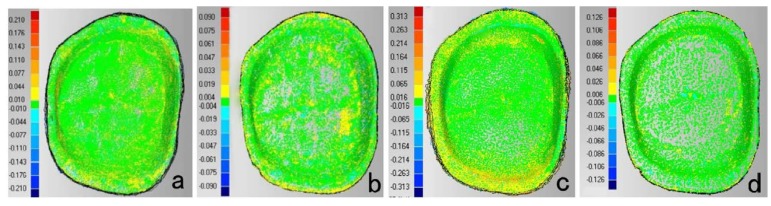
Color-coded difference images showed the precision of each scanner. (**a**) TRIOS (**b**) D700 (**c**) MHT (**d**) inEos (Buccal surface is on the top; Mesial surface is on the right).

According to the results of this study, color-coded difference images (Figure 1) implied qualitative information. For TRIOS, greater deviations mainly lay on buccal and lingual margin, distal surfaces and corners. For MHT, deviations mainly lay on lingual margin, distal surface, and corners. It was reported that when obvious angles (>60°) exist between the perpendicular of scanned surface and the scanning direction, the accuracy of digitization declined [26]. Studies [9] also pointed out that proximal surfaces’ scanning was the difficulty of digital impression-taking. In order to get the intact images of margin and proximal surfaces (especially the distal surface) under simulated intraoral conditions, the scanning head must be adjusted into several directions to get rid of blocking of adjacent teeth. Deviations around corners approved of Rudolph’s discovery [13] that tooth shape was a dominating factor for precision and that large deviations occurred in areas with strong changes of curvature and steep surface [27]. In this study, D700 and inEos model images showed a relative uniform distribution pattern compared to digital impressions, because D700 and inEos could scan the preparation die separately to get rid of the blocking of adjacent tissues.

**Table 1:** RMS analysis of precision for each scanner (μm).

Group	Maximum	Mean	Median	Interquartile Range
TRIOS	9.72	7.62	7.75	1.34
D700	5.53	4.47	4.45	1.32
MHT	19.69	12.49	12.00	3.80
inEos	4.66	3.35	3.14	0.77

Significant differences were found between scanners (*p* < 0.001).

### 3.2. Trueness

To receive a REF, several approaches were described [16,28,29], such as applying a coordinate measuring machine (CMM), computed tomography (CT) and an optical technology based on focus variation. In this study, the preparation was digitized by micro-CT to obtain a highly accurate reference dataset due to micro-CT exhibited higher accuracy than the tested dental scanners.

Table 2 gives the analyses of RMS deviations between REF and each scanner. TRIOS (64.25 ± 3.45 μm) and MHT (61.89 ± 3.45 μm) showed the comparable least deviations, followed by D700 (66.68 ± 0.85 μm) and inEos (71.19 ± 1.70 μm). Digital impressions were found to have better trueness than model images, which was consistent with Güth’s study about four-unit fixed-partial dentures [12]. Based on the results of this study and previous studies about trueness [16,30,31], we could draw the conclusion that digital impressions did better in short dentition scanning, but still needed to be improved for full-arch scanning.

**Figure 2:**
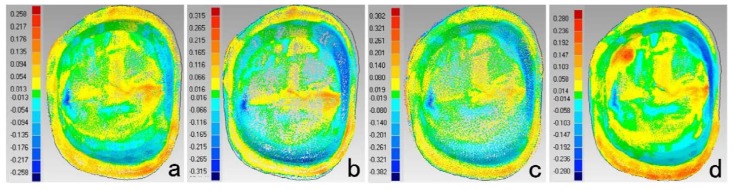
Color-coded difference images showed the deviations between REF and each scanner. (**a**) TRIOS (**b**) D700 (**c**) MHT (**d**) inEos (Buccal surface is on the top; mesial surface is on the right.)

Figure 2 displayed the color-coded difference images between REF and each scanner. Digital impressions showed greater positive deviations along the marginal area, which was in accordance with the results of precision. It could be concluded that lower accuracy occurred in marginal areas of digital impressions. Greater deviations on mesial and lingual surfaces were found for model scanner, might due to the volume changes of impressions and casts. Nassar [4] found that shrinkage of impression materials occured toward the tray wall in buccolingual direction. The Aquasil impression materials reported a shrinkage of 0.06%–0.24% [7,32] mainly in the buccallingual and mesialdistal direction [7], while an expansion of 0.20% in occlusalgingival direction [7]. Moreover, expansion of the type IV dental stone was reported to be 0.14% to 0.21% [5], which increased the changes in axial directions but compensated the changes in the occlusalgingival direction. So smaller deviations were found on occlusal surface for model scanners.

**Table 2:** Analyses of RMS between REF and each scanner (μm). SD is standard deviation. CI is confidence interval.

Group	Mean ± SD	95% CI	
		Low	High
TRIOS *	64.25 ± 3.45	61.78	66.72
D700	66.68 ± 0.85	66.06	67.29
MHT *	61.89 ± 3.45	59.43	64.36
inEos	71.19 ± 1.70	69.29	73.03

* No significant difference was found between MHT and TRIOS (*p* > 0.05).

**Figure 3:**
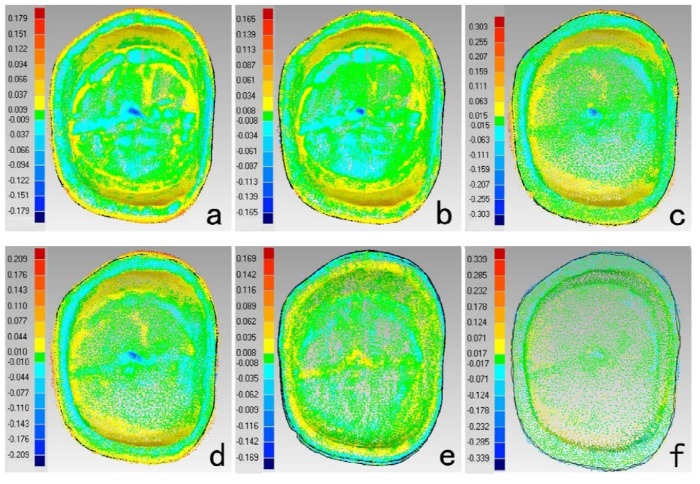
Color-coded difference images showed the deviations between scanners. (**a**) TRIOS-D700 (**b**) TRIOS-inEos (**c**) MHT-inEos (**d**) MHT-D700 (**e**) inEos-D700 (**f**) TRIOS-MHT inEos (Buccal surface is on the top; Mesial surface is on the right.)

The results of deviations among scanners were showed in Table 3 and Figure 3. The deviations between digital impressions and model images were mainly in buccal and lingual surfaces and occlusal fossa. The deviations distributions of TRIOS-MHT and inEos-D700 were relatively uniform. InEos-D700 had nice consistency (RMS < 10 μm), which also demonstrated the lower trueness of D700 and inEos might be due to the errors accumulated during the traditional impression and stone cast procedures. Higher deviations on buccal and lingual surfaces between digital impression and model images might be due to the changes of impressions and stone casts, while the deviations on proximal surfaces were counteracted. However, no direct comparison between intraoral and model scanners without the errors of impression-taking and stone cast-making process was the limitation of this study.

**Table 3:** Statistical analyses of RMS between groups (μm).

Group	TRIOS-D700	TRIOS-inEos	MHT-inEos	MHT-D700	inEos-D700	TRIOS-MHT
mean	16.764	16.285	21.245	21.662	8.534	16.523
SD	1.27	1.31	2.80	2.75	0.89	1.92

### 3.3. Marginal and Internal Fit

Several strategies have been introduced to detect fitness, which are directly view the marginal gap, cross-section, replica-technique, and micro-CT analysis [33]. Although cross-section could only provide 2-dimensional information of the adhesive gap, it could best mimic the complex clinical procedures.

**Figure 4:**
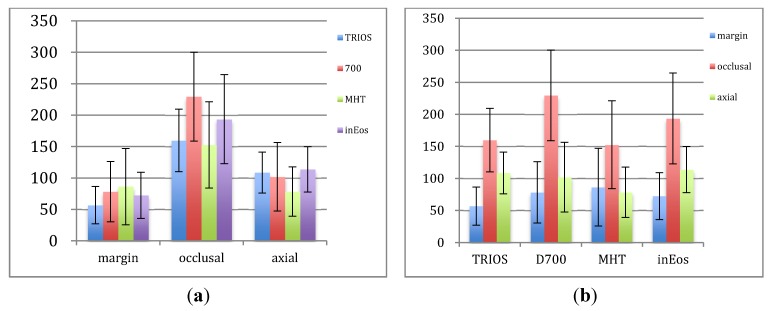
Comparison of the means from each group of marginal, occlusal and axial gaps (μm). (**a**) Comparison of different groups for each location; (**b**) Comparison of different locations for each group.

Figure 4 showed the mean values of MG, IGO and IGA. In our cross-section test compared to the trueness, the adhesive gaps were much higher for all groups. There was a tendency that IGO > IGA > MG in TRIOS, D700 and inEos (*p* < 0.01), while MG and IGA values were smaller than IGO in MHT (*p* < 0.01). In this study, the following factors should be taken into consideration: (1) in the design of the crowns, the simulated cement thickness was set at 60 μm, starting 1 mm from the finishing line, (2) cementation could cause an increase of the adhesive gap have been reported in both *in vitro* studies [34] and clinical studies [35].

For MG, TRIOS was smaller than MHT (*p* < 0.01). Digital impressions showed a relative comparable MG to indirect methods, meanwhile, all the Mean values and 95% confidence intervals of MG met the clinical standard (lower than 120 μm) according to Mclean’s [21] recommendation, which might demonstrate that lower accuracy on margins of digital impressions couldn’t cause clinical difference. MHT and TRIOS showed smaller IGO than inEos and D700. MHT showed significantly lower IGA than the others (*p* < 0.01), while no statistical significant differences of IGA were found among TRIOS, D700 and inEos. These were in accordance with our above-mentioned results, that digital impressions had better trueness than model images. The similar gap distribution and discrepancy in D700 and inEos was also in accordance with the result of inter-group comparison that inEos-D700 had nice consistency. TRIOS showed lower MG and higher IGA than MHT but comparable IGO, which demonstrated the results that TRIOS and MHT had similar trueness while TRIOS-MHT comparison didn’t show nice consistency.

Mean values of MG, IGO and IGA of distal (D), mesial (M), buccal (B) and lingual (L) surfaces in our test were showed in Table 4. Since normality and equality assumptions were met, one-way ANOVA combined with LSD post hoc test showed that only IGA of TRIOS and D700 and MG of MHT showed significant differences between different surfaces, which is similar to Syrek A’s discovery [36]. No regular pattern was found about the gap distribution in different surfaces, which may be due to the disturbance of cementation procedure. Further studies are still needed to explore the effect of cementation on fitness in different surfaces.

Although several previous studies [36,37] have evaluated the IG (IGA and IGO) and MG of restorations based on digital impressions, no consistent conclusions have been drawn. Based on the limited results of our study, the preliminary conclusion could be drawn that the fitness of restorations based on digital impressions was up to clinical standards. Digital impression could be an alternative to conventional impression technique for single crown manufacturing.

**Table 4:** Comparison of the marginal, occlusal and axial gaps in distal, mesial, buccal, and lingual surfaces of each group (μm).

Group	Surface	MG	IGO	IGA
TRIOS	D	62.96 ± 30.38	140.87 ± 35.89	85.15 ± 27.03 ^1^
	M	56.29 ± 32.44	179.94 ± 55.86	112.79 ± 38.96 ^1^
	B	66.49 ± 29.71	153.89 ± 29.06	93.50 ± 20.73 ^1^
	L	40.41 ± 22.75	164.38 ± 67.22	132.86 ± 14.14 ^1^
	Mean	56.78 ± 29.75	159.77 ± 49.65	108.58 ± 32.65
D700	D	82.76 ± 33.54	204.60 ± 50.74	101.00 ± 52.44 ^2^
	M	103.02 ± 76.82	211.83 ± 62.51	139.44 ± 70.83 ^2^
	B	71.98 ± 32.79	250.90 ± 99.74	97.33 ± 38.59 ^2^
	L	55.71 ± 23.20	250.98 ± 56.36	70.53 ± 30.16 ^2^
	Mean	78.37 ± 47.92	229.58 ± 70.71	102.08 ± 54.45
MHT	D	128.68 ± 73.95 ^3^	164.26 ± 24.46	74.04 ± 42.52
	M	88.49 ± 72.77 ^3^	144.32 ± 79.75	77.69 ± 46.11
	B	70.65 ± 32.56 ^3^	164.90 ± 87.46	89.27 ± 36.15
	L	57.62 ± 30.46 ^3^	136.93 ± 71.36	72.73 ± 32.25
	Mean	86.36 ± 60.65	152.60 ± 68.56	78.43 ± 39.24
inEos	D	66.97 ± 19.70	170.55 ± 56.87	128.95 ± 38.95
	M	81.09 ± 54.37	235.78 ± 63.83	118.13 ± 36.85
	B	78.93 ± 39.89	171.89 ± 85.47	101.36 ± 28.82
	L	62.65 ± 25.24	196.23 ± 63.99	106.87 ± 38.03
	Mean	72.41 ± 36.72	193.61 ± 70.94	113.83 ± 36.12

^1^, ^2^ and ^3^ indicated significant differences (*p* < 0.05).

## 4. Conclusions

Within the limitations of this study, the following conclusions could be drawn:

(1) Digital impressions showed better trueness and lower precision compared to model images. Lower accuracy occurred in marginal areas of digital impressions.

(2) Marginal discrepancies in all groups were up to clinical standard. Digital impressions showed better occlusal fitness.

(3) Digital impression could be an alternative to conventional impression for single crown manufacturing.
